# Micafungin versus caspofungin in the treatment of *Candida glabrata* infection: a case report

**DOI:** 10.1186/s13256-016-1096-z

**Published:** 2016-11-08

**Authors:** Shoko Merrit Yamada, Yusuke Tomita, Tomotsugu Yamaguchi, Toshiaki Matsuki

**Affiliations:** 1 Department of Neurosurgery, Teikyo University Mizonokuchi Hospital, 3-8-3 Mizonokuchi, Takatsu-ku, Kawasaki, Kanagawa 213-8507 Japan; 2 Department of Pharmacy, Teikyo University Mizonokuchi Hospital, 3-8-3 Mizonokuchi, Takatsu-ku, Kawasaki, Kanagawa 213-8507 Japan

**Keywords:** Candidiasis, Caspofungin, Echinocandins, Fungal infection, Micafungin, Case report

## Abstract

**Background:**

Micafungin and caspofungin, which are both echinocandins, elicit their antifungal effects by suppressing the synthesis of β-D-glucan, an essential component of fungal cell walls. If micafungin is not effective against a fungal infection, is it unreasonable to switch to caspofungin?

**Case presentation:**

An 80-year-old Asian man presented to our hospital with brain and lung abscesses. *Klebsiella pneumonia* and *Escherichia coli* were identified by sputa culture and *Streptococcus mitis* was identified in the brain abscess culture obtained by drainage surgery. He was treated with antibiotics and both abscesses shrank after the treatment. But he continued to have a high fever and *Candida glabrata* was identified by blood culture. The origin of the infection was not clarified and micafungin was administered intravenously. The fungus showed poor susceptibility to micafungin; we then switched the antifungal from micafungin to caspofungin. After caspofungin treatment, his body temperature remained below 37 °C and his β-D-glucan levels decreased remarkably.

**Conclusions:**

*In vitro*, micafungin is considered more effective against *C. glabrata* because its minimum inhibitory concentration against *C. glabrata* is lower than that of caspofungin. However, *in vivo*, there is no significantly different effect between the two drugs. When micafungin is not effective against candidiasis, a switch to caspofungin might be applicable because the pharmacokinetics in each echinocandin is slightly different.

## Background

Antifungal agents that are currently used for deep fungal infections include polyene macrolide derivatives (amphotericin B), azole antifungals (imidazoles and triazoles), and echinocandins. Echinocandins are recommended as the primary therapy for invasive candidiasis [1], and resistance to echinocandins remains low, at less than 3 %, in *Candida albicans* [2]. However, cases of echinocandin-resistant *Candida glabrata* are increasing with cross-resistance to azole antifungal agents [3, 4]. Here, we report a case of successful treatment for *C. glabrata* infection that showed poor sensitivity to micafungin (MCFG), which is an echinocandin.

## Case presentation

**Fig. 1:**
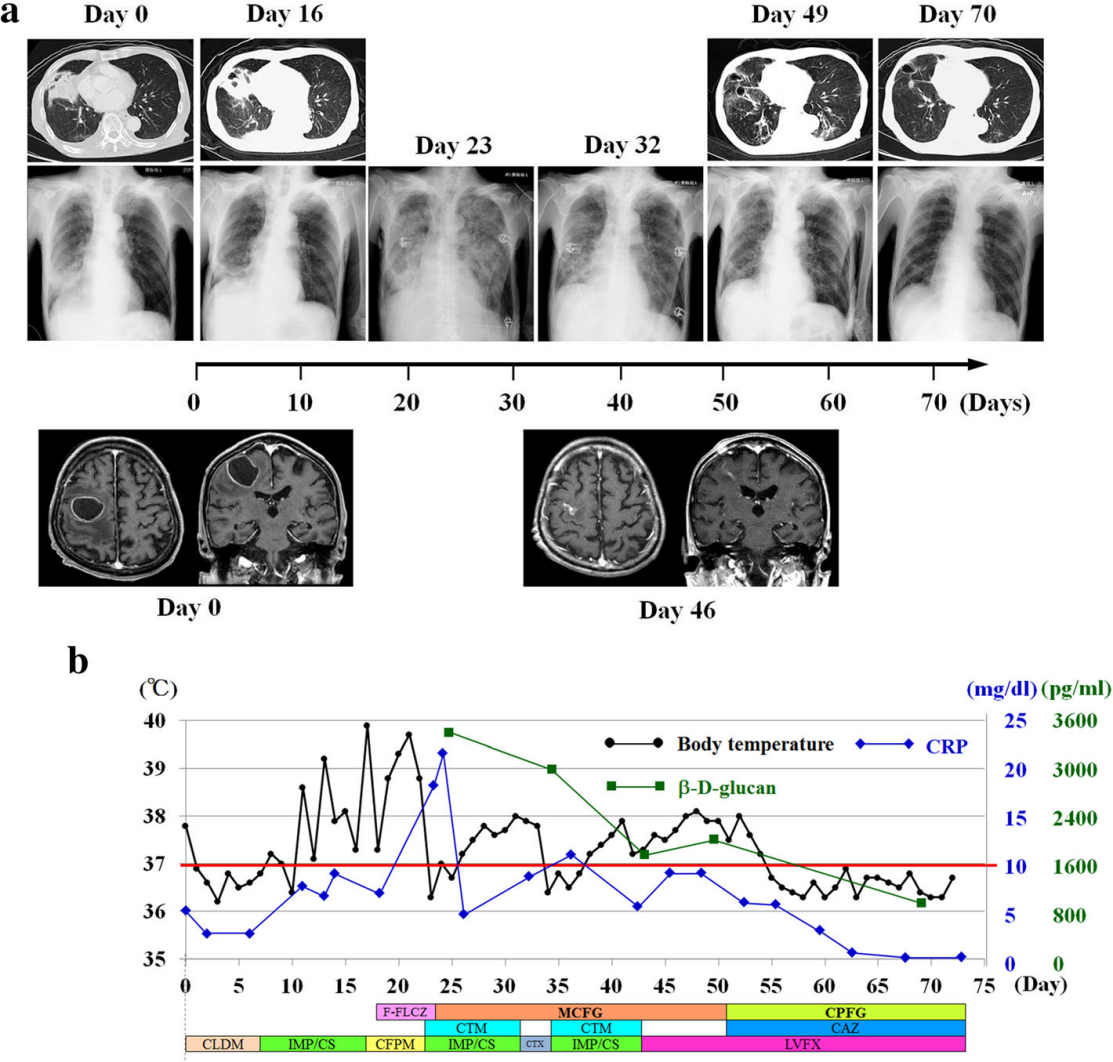
Chronological changes in the brain and lung abscesses. **a** Image studies: *top*, chest computed tomography scan; *middle*, chest X-ray; and *bottom*, head magnetic resonance imaging with contrast medium. **b** Alterations in body temperature (*black*), C-reactive protein levels (*blue*), and β-D-glucan levels (*green*) in response to the changes in antibiotics and antifungals. Daily doses: clindamycin 600 mg, twice a day; imipenem/cilastatin sodium 0.5 g, twice a day; cefepime dihydrochloride hydrate 1 g, twice a day; cefotiam hydrochloride 1 g, twice a day; levofloxacin hydrate 500 mg, once a day; ceftazidime hydrate 1 g, twice a day; fosfluconazole 800 mg, once a day on the first day, and 400 mg, once a day after second day; micafungin sodium 150 mg, once a day; and caspofungin acetate 70 mg, once a day on the first day, and 50 mg, once a day after second day. The *red* horizontal line in the graph indicates the body temperature level of 37 °C and CRP level of 10 mg/dl. *CAZ* ceftazidime hydrate, *CFPM* cefepime dihydrochloride hydrate, *CLDM* clindamycin, *CPFG* caspofungin acetate, *CRP* C-reactive protein, *CTM* cefotiam hydrochloride, *CTX* cefotaxime, *F-FLCZ* fosfluconazole, *IMP/CS* imipenem/cilastatin sodium, *LVFX* levofloxacin hydrate, *MCFG* micafungin sodium

An 80-year-old Asian man presented to our hospital with left hemiparesis; a manual muscle test revealed motor weaknesses of 2+/5 in his upper limbs and 4/5 in his lower limbs. Magnetic resonance imaging (MRI) demonstrated a brain abscess in his right frontal lobe (motor area), and both a chest X-ray and a computed tomography (CT) scan showed a lung abscess in the lower lobe of his right lung (Fig. 1a, day 0). He had undergone partial resection of his stomach due to gastric cancer 11 years ago, but he did not take anti-cancer drugs and no recurrence was identified. The drugs he had been taking were 50 mg of sodium ferrous citrate, 1500 μg vitamin B12, 20 mg esomeprazole, and 2.5 mg amlodipine. His medical history did not indicate an immune deficiency disorder. No organ dysfunction was indicated from laboratory data. But mild anemia was identified: his hemoglobin value was 11.9 g/dl (normal range 13.5 to 17.6), hematocrit 35.0 % (39.8 to 51.8), iron (Fe) 49 mg/dl (50 to 170), and ferritin 98.9 ng/ml (21 to 282). Because he was not able to eat enough food for 1 week before admission, his nutritional status was poor: serum total protein 6.1 g/dl (normal range 6.7 to 8.3), albumin 3.2 g/dl (3.8 to 5.1), total cholesterol 148 mg/dl (150 to 219), and triglyceride 126 mg/dl (50 to 150).

### Days 0–10

On admission, he was administered clindamycin (CLDM; 600 mg twice a day) because we were considering aspiration pneumonia. However, this was switched to imipenem/cilastatin sodium (IMP/CS; 0.5 g twice a day) on day 6 post-admission because *Klebsiella pneumoniae* and *Escherichia coli* were identified by sputa culture (Fig. 1b).

### Days 11–20

After the antibiotic was changed to IMP/CS, his body temperature gradually elevated, his serum C-reactive protein (CRP) level also increased to 7.8 mg/dl (normal range <0.3 mg/dl), and his left hemiparesis worsened. To address these symptoms, a surgical aspiration of his brain abscess was performed under local anesthesia on day 12. Although his left hemiparesis mildly improved after the surgery and the follow-up chest X-ray and CT scan showed that he was recovering from pneumonia (Fig. 1a, Day 16), high fever continued after the surgery in spite of the IMP/CS treatment. *Streptococcus mitis* was identified in the brain abscess culture, and a blood culture performed on day 16 indicated he had a simultaneous fungal infection. The antibiotic was changed to cefepime dihydrochloride hydrate (CFPM; 1 g twice a day) targeting a Gram-positive coccus and fosfluconazole (F-FLCZ; 800 mg once a day on the first day and 400 mg once a day after second day) treatment was added for fungal infection. Despite these treatments, he still had a high body temperature (39.7 °C) on day 20 (Fig. 1b). At this time, a central venous catheter was inserted in his right subclavian vein for parenteral nutrition because he was unable to get sufficient nutrients through oral intake.

### Days 21–30

On day 21, 4 days after CFPM treatment started, his body temperature elevated again to 39.6 °C and his serum CRP level drastically increased. He had difficulty in breathing and was intubated with artificial respirator assistance because his chest X-ray showed diffuse interstitial pneumonia (Fig. 1a, Day 23) and *E. coli* was still identified in sputa culture. The antibiotic was switched back to IMP/CS (0.5 g twice a day) targeting *E.coli*, and cefotiam hydrochloride (CTM; 1 g twice a day) was used for brain abscess treatment because we considered it had good transitivity of the antibiotic to the cerebrospinal fluid. In addition, *C. glabrata*, which is resistant to F-FLCZ, was detected via the blood culture performed on day 16, and his level of β-D-glucan was extremely high at 3634 pg/ml (normal range <20 pg/ml). Then, he received combination treatments of MCFG (150 mg once a day), IMP/CS (0.5 g twice a day), and CTM (1 g twice a day) because of multiple infections resulting from fungi and from both Gram-negative bacillus and Gram-positive coccus bacteria (Fig. 1b).

### Days 31–50

A chest X-ray on day 32 displayed an improvement in his diffuse interstitial pneumonia (Fig. 1a), and he was extubated. His antibiotic treatments with IMP/CS and CTM were changed to cefotaxime (CTX; 1 g twice a day) because two strong antibiotics were used for 1 week. However, his CRP rose rapidly to 7.4 mg/dl from 4.7, and the antibiotic was switched back to IMP/CS (0.5 g twice a day) and CTM (1 g twice a day). Another 1 week of combination treatment of IMP/CS and CTM was performed. His body temperature was constantly elevated over 37.0 °C and his CRP never decreased less than 5.0 mg/dl. Then, levofloxacin hydrate (LVFX; 500 mg once a day), which was classified as quinolone antibiotic, was applied from day 43. During the period of MCFG administration, his body temperature fluctuated between 37 and 38 °C and his CRP level varied between 5 and 10 mg/dl. On day 43, his level of β-D-glucan decreased to 1732 pg/ml. MRI on day 46 revealed that his brain abscess had almost completely disappeared (Fig. 1a, day 46) and a chest X-ray demonstrated amelioration of his pneumonia (Fig. 1a, Day 49). However, on day 49, his body temperature climbed to 38 °C, his CRP level increased to 9.8 mg/dl, and his β-D-glucan level elevated to 1947 pg/ml. To address these symptoms, on day 51, the MCFG was switched to caspofungin (CPFG; 70 mg once a day on the first day and 50 mg once a day after second day) and ceftazidime hydrate (CAZ; 1 g twice a day) treatment was added (Fig. 1b).

### Days 51–72

Three days after initiating the CPFG and CAZ treatments, his body temperature began decreasing and never exceeded 37 °C after day 55. His CRP level also rapidly decreased after the initiation of CPFG treatment; it dropped within the normal range and did not increase again after day 63. The central venous catheter was removed on day 65 because he could ingest fully. His β-D-glucan level decreased to 1000 pg/ml (Fig. 1b), and *C. glabrata* was not identified in the blood culture performed on day 69.

At day 70, a chest X-ray and CT scan demonstrated successful treatment of his lung abscess and pneumonia (Fig. 1a). When he was discharged from our hospital on day 73, he had regained his motor function (4−/5 in his left upper limb and 5/5 in his left lower limb by a manual muscle test).

## Conclusions

In our patient, the risk factors for candidemia included partial resection of stomach causing Fe deficiency anemia, malnutritional status on admission, age, and the long-term usage of antibiotics. *C. glabrata* is one of the prevalent causes of candidemia, and it exhibits poor susceptibility to azole antifungals, acquiring resistance rapidly [5, 6]. Instead, echinocandins are recommended when *Candida* infection is suspected [1]. Anidulafungin (ANFG), CPFG, and MCFG are all echinocandins with similar antifungal activity spectra [7–9], but only CPFG and MCFG are authorized in Japan. In *in vitro* studies, the minimum inhibitory concentration against *C. glabrata* was higher in CPFG than in ANFG or MCFG, and MCFG was the most potent agent against *C. glabrata* [9–11]. However, the clinical outcomes of patients with systemic candidiasis due to *C. glabrata* were not significantly different if they were treated with MCFG or with CPFG [12].

The level of serum β-D-glucan increased in spite of MCFG treatment in our patient; however, we switched the antifungal to CPFG, another echinocandin, rather than changing to a different class of antifungals because echinocandins suppress β-D-glucan synthesis better than any other antifungals [13]. Notably, CPFG treatment was dramatically effective in our case. Spreghini *et al*. found that, in the absence of human serum, the susceptibilities of *C. glabrata* to MCFG and to CPFG were not statistically different from one another, whereas, in the presence of human serum, *C. glabrata* was more susceptible to CPFG than to MCFG (*p* <0.05) [14]. In their study using mice, CPFG started to be effective after 2 days of treatment, while after 6 days, the lowest effective doses were 0.25, 1, and 5 mg/kg/day for CPFG, MCFG, and ANFG, respectively [14]. Our case clearly demonstrates the effectiveness of CPFG in a patient with *C. glabrata* infection that showed resistance to MCFG. In echinocandin pharmacokinetics, the protein-binding form of the drug constitutes 99.8 % in MCFG and 97 % in CPFG, and the clearance of CPFG from the human body is much slower than that of MCFG [13]. The lower percentage of protein-binding form and slower excretion of CPFG may help it maintain an effective drug concentration against *C. glabrata* for longer than MCFG.

In fact, invasive candidiasis was successfully treated by CPFG in our case, but we do not conclude that CPFG is more effective than MCFG for *C. glabrata* infection because the *in vivo* antifungal activity, pharmacokinetics, and toxicity profiles are slightly different in each echinocandin [15]. We considered that the sensitivity pattern of the *Candida* to echinocandins in our patient was that the *C. glabrata* in our patient was basically sensitive to echinocandins, and it was more sensitive to CPFG than to MCFG. Some advantages of MCFG treatment are that serious side effects rarely occur and it can be used when the patient has mild to severe hepatic dysfunction [13, 16]. Unfortunately, CPFG is the only echinocandin for which dosage reduction is recommended if patients have even mild hepatic dysfunction [1]. Therefore, MCFG should still be the first drug choice for candidemia; however, if it is not effective, early replacement with CPFG should be considered, rather than adding another kind of antifungal agent.

## Abbreviations

ANFG: Anidulafungin
CAZ: Ceftazidime hydrate
CFPM: Cefepime dihydrochloride hydrate
CLDM: Clindamycin
CPFG: Caspofungin
CRP: Serum C-reactive protein
CT: Computed tomography
CTM: Cefotiam hydrochloride
CTX: Cefotaxime
Fe: Iron
F-FLCZ: Fosfluconazole
IMP/CS: Imipenem/cilastatin sodium
LVFX: Levofloxacin hydrate
MCFG: Micafungin
MRI: Magnetic resonance imaging
